# Nursing-based precision assessment of nutrition and muscle status without ultrasound: a prospective observational study in mechanically ventilated neurosurgical patients

**DOI:** 10.3389/fmed.2025.1737401

**Published:** 2026-01-14

**Authors:** Chenliang Pan, Dingding Xu, Zixin Wang, Jia Wen, Lili Ma, Yajuan Zhang

**Affiliations:** 1 Department of Nursing, Shanghai Sixth People’s Hospital Affiliated to Shanghai Jiao Tong University School of Medicine, Shanghai, China; 2 Department of Nursing, Shanghai General Hospital, Shanghai Jiao Tong University School of Medicine, Shanghai, China; 3 Department of Nursing, Shanghai East Hospital, School of Medicine, Tongji University, Shanghai, China

**Keywords:** diaphragm thickening fraction, mechanical ventilation, mNUTRIC score, NICU, nursing assessment

## Abstract

**Background:**

Critically ill neurosurgical patients on prolonged mechanical ventilation rapidly develop muscle wasting and malnutrition that hinder weaning and recovery. Ultrasound is useful but not always available for routine nursing assessment. We evaluated whether nurse-led, routinely available indicators can reflect muscle status and complement ultrasound monitoring.

**Methods:**

We conducted a single-center prospective observational study in one neurosurgical intensive care unit (NICU) from January to October 2025. Adults expected to remain in ICU for at least 7 days and on mechanical ventilation for at least 7 days were enrolled; 137 patients were analyzed. Nutrition-related indicators included the mNUTRIC score, serum albumin, and total protein. Muscle-related measures were recorded on ICU days 1, 3, and 7 and included quadriceps and rectus femoris thickness or cross-sectional area, the Medical Research Council score, diaphragm thickness at end-expiration and end-inspiration, diaphragm thickening fraction, diaphragmatic excursion, gastrocnemius indices, and bilateral lower-limb circumference.

**Results:**

During the first ICU week, peripheral muscle parameters—quadriceps, rectus femoris, and gastrocnemius—and respiratory muscle parameters—diaphragm thickening fraction, diaphragmatic excursion, and diaphragm thickness—declined significantly, consistent with early muscle wasting under mechanical ventilation. Nutritional risk increased in parallel, with decreases in albumin and total protein. The mNUTRIC score showed significant negative correlations with 13 muscle-related parameters, and albumin and total protein were positively correlated with diaphragm thickening fraction and lower-limb circumference (all *p* < 0.05).

**Conclusion:**

In NICU patients ventilated for at least 7 days, muscle mass and diaphragm function deteriorate early and accompany rising nutritional risk. Nurse-led indicators—mNUTRIC, albumin, and total protein—provide pragmatic screening for muscle wasting and can flag patients needing intensified nutrition or rehabilitation, while ultrasound remains indispensable for high-precision decisions such as weaning evaluation. A tiered assessment strategy may enhance applicability across ICUs with diverse resources.

## 1 Introduction

Critically ill neurosurgical patients frequently require prolonged mechanical ventilation due to traumatic brain injury, intracerebral hemorrhage, or postoperative complications, and ventilation often extends beyond 7 days in complex cases. Although life-sustaining, long-term ventilation contributes to muscle inactivity, hypercatabolism, and increased energy expenditure, accelerating muscle loss and functional decline (1, 2). Feeding intolerance, impaired gastrointestinal function, and stress responses further reduce nutritional intake, increasing the risk of malnutrition (3), which in turn exacerbates respiratory muscle weakness and negatively affects spontaneous breathing trials and rehabilitation outcomes (4).

Bedside ultrasound has emerged as a noninvasive tool for evaluating muscle and respiratory function, including quadriceps thickness, rectus femoris area, diaphragm thickness, diaphragm thickening fraction (DTF), and diaphragmatic excursion (5, 6). Muscle strength assessment, such as the Medical Research Council (MRC) score, provides standardized functional evaluation (7). Nutritional assessments—including the mNUTRIC score and serum albumin and total protein levels—offer additional information on nutritional risk and status (3, 8). However, few studies have examined the relationship between nutritional status and muscle loss specifically in neurosurgical patients requiring prolonged ventilation (9), and most available data come from general ICU populations with short follow-up windows (10).

Despite its precision, ultrasound implementation is limited by operator dependency, training requirements, and equipment availability (11). In contrast, mNUTRIC scoring and serum markers are simple, inexpensive, and widely accessible (12). If shown to correlate with muscle wasting (13), these routine indicators may help clinicians identify high-risk patients earlier and integrate nutritional, respiratory, and rehabilitation strategies in a timely manner (14).

This study evaluated longitudinal changes in nutritional and muscle-related parameters on ICU days 1, 3, and 7 to clarify associations and support improved clinical management in NICU patients receiving mechanical ventilation ≥7 days.

## 2 Methods

### 2.1 Study design and participants

This was a single-center, prospective observational study conducted in a NICU from January to October 2025. The study population included adult patients receiving mechanical ventilation during their NICU stay. Inclusion criteria were as follows: (1) age ≥18 years; (2) admission to the neurosurgical intensive care unit; (3) duration of mechanical ventilation ≥7 days; (4) expected ICU stay ≥7 days; and (5) provision of written informed consent by the patient or their legal representative. Exclusion criteria included: (1) pre-existing severe neuromuscular diseases (e.g., muscular dystrophy, polymyositis, myasthenia gravis) or baseline conditions affecting muscle status (e.g., thyroid disorders, advanced cachexia from malignancy); (2) long-term enteral or parenteral nutritional support prior to admission; (3) severe lower-limb edema (defined as pitting edema ≥ + 2), limb amputation, or other conditions preventing accurate muscle measurement by ultrasound; (4) pregnancy or lactation; (5) Receipt of high-dose systemic corticosteroids (equivalent to >20 mg/day of prednisone) for >7 days prior to ICU admission or during the study period. Post-hoc exclusion criteria was patients who were extubated, transferred, or died before day 7.

**Figure 1:**
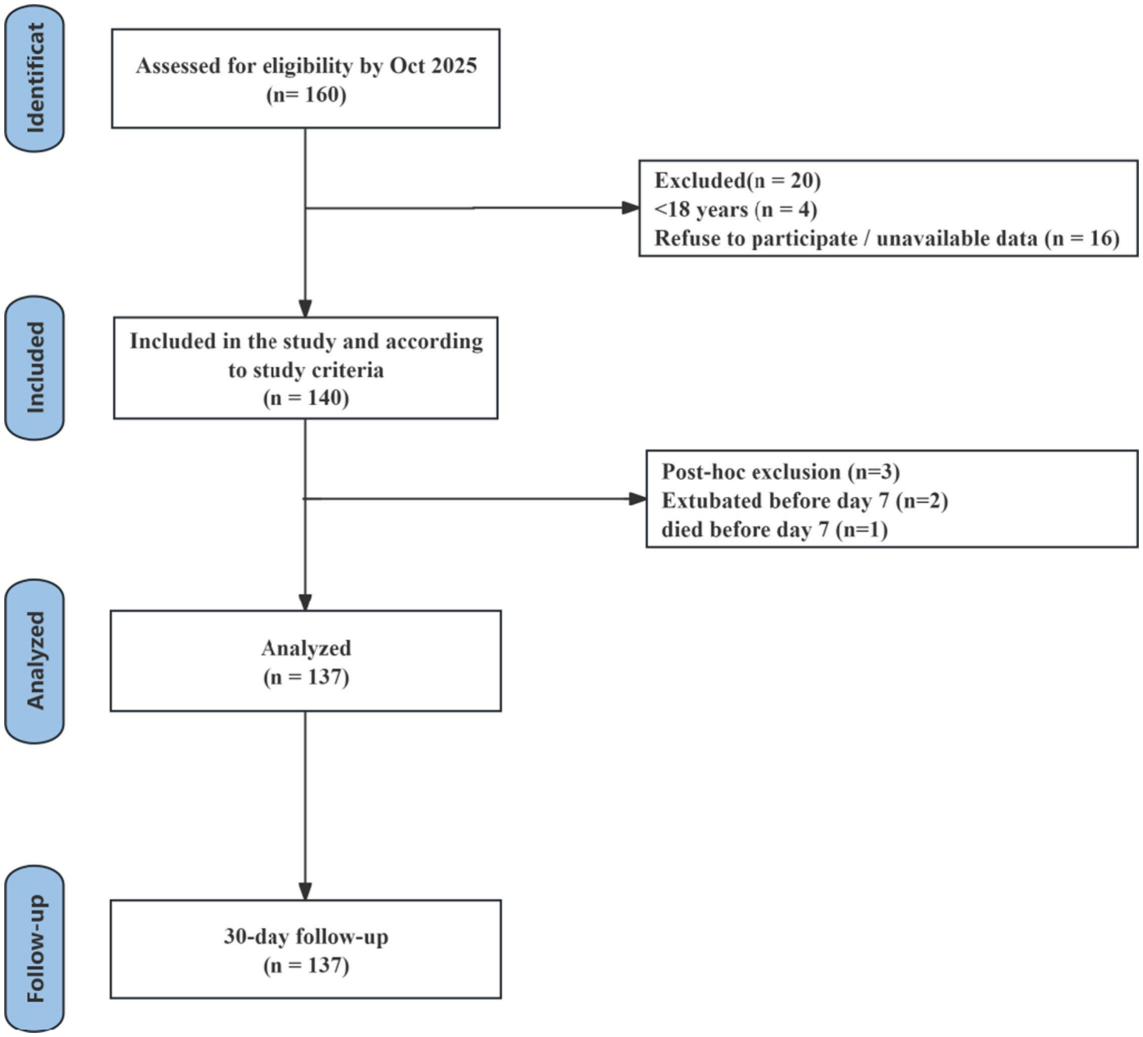
The participant flow diagram.

One hundred forty participants were included in the study. One patient died, and two patients extruded before day 7. The complete participant flow diagram is shown in Figure 1. A total of 137 patients met the eligibility criteria and were included in the final analysis. All patients received enteral or parenteral nutritional support according to the ICU standard protocol, with an energy target of 25–30 kcal/kg/day. To enable the scheduled longitudinal assessments, patients were required to have an expected ICU stay of ≥7 days at enrollment. Follow-up assessments were performed on days 1, 3, and 7 after enrollment. Thirty-day outcomes (e.g., mortality and extubating status) were ascertained from the hospital electronic medical record system.

According to the sample size calculation formula for multivariate analysis, *n* = 1 + *m* + *mψ*^2^ (1/*R*^2^–1). In this study, the number of independent variables was *m* = 6 (including four ultrasound muscle dimensions and two nutritional adequacy dimensions). With a two-sided test at a significance level of *α* = 0.05, *ψ* = 1.9661. Based on the linear regression analysis from the pilot study, R was estimated to be 0.401. The calculated sample size was therefore *n* = 1 + 6 + 6 × 1.9661^2^ × (1/0.401^2^–1) ≈ 127. Considering a 20% attrition or invalid response rate, the final required sample size was determined to be 152 participants.

### 2.2 Data collection and measurement variables

#### 2.2.1 Baseline characteristics

The following baseline data were collected: age, sex, body mass index (BMI), Acute Physiology and Chronic Health Evaluation II (APACHE II) score, Sequential Organ Failure Assessment (SOFA) score, primary diagnosis, clinical outcome (transfer to rehabilitation, treatment withdrawal, or discharge against medical advice), and achievement of nutritional and rehabilitation targets on ICU days 3 and 7.

##### 2.2.1.1 APACHE II score

The APACHE II system evaluates disease severity in critically ill patients and comprises three components: acute physiology, age, and chronic health condition. Scores range from 0 to 71, with higher scores indicating greater severity (15). Two trained research nurses calculated the APACHE II score using physiological data, laboratory results, and clinical history within the first 24 h after ICU admission.

##### 2.2.1.2 SOFA score

The SOFA score reflects the degree of multiple organ dysfunction across six systems: respiratory, coagulation, hepatic, cardiovascular, central nervous, and renal (16). Each organ is scored from 0 to 4, with a total score ranging from 0 to 24, where higher scores represent more severe organ dysfunction (16). Certified critical care nurses from the research team independently assessed the SOFA score based on the first-day laboratory and clinical data.

##### 2.2.1.3 Primary diagnosis

The primary diagnosis was determined from admission records and included conditions such as intracerebral hemorrhage, cerebral infarction, traumatic brain injury, and brainstem lesions.

##### 2.2.1.4 Clinical outcome

Outcomes were classified according to discharge summaries in the electronic medical record system as (a) improved and transferred to a rehabilitation ward, (b) treatment withdrawal and transfer, or (c) discharge against medical advice.

##### 2.2.1.5 Nutritional and rehabilitation target achievement

Target attainment on ICU days 3 and 7 was evaluated by the research team according to predefined criteria, including attainment of energy intake goals and completion of basic rehabilitation training tasks.

All data were obtained from the hospital electronic medical record system, nursing documentation, and laboratory information systems. Data extraction was performed independently by two trained research nurses. In cases of discrepancy, a third investigator reviewed the records to ensure data accuracy and consistency.

#### 2.2.2 Muscle-related parameters

Ultrasound examinations were performed using a Mindray M9 system. A high-frequency linear array probe (10–15 MHz) was used for quadriceps, rectus femoris, gastrocnemius, and diaphragm thickness measurements. A low-frequency phased array probe (2–5 MHz) was used for diaphragmatic excursion measurement. Muscle-related parameters were collected on ICU day 1 (D1), day 3 (D3), and day 7 (D7), including quadriceps muscle thickness, rectus femoris thickness and cross-sectional area, diaphragm end-expiratory thickness (DTe), end-inspiratory thickness (DTi), diaphragm thickening fraction, diaphragm excursion (DE), gastrocnemius thickness, gastrocnemius pennation angle, bilateral calf circumference, and lower-limb muscle strength assessed by the MRC score.

##### 2.2.2.1 Ventilator settings

All diaphragm ultrasound measurements were performed while patients were under stable mechanical ventilation. The ventilator mode was set to assist/control (A/C) with a tidal volume (VT) of 6–8 mL/kg predicted body weight, a positive end-expiratory pressure (PEEP) of 5 cm H_2_O, and the fraction of inspired oxygen (FiO_2_) maintained at the lowest level required to achieve target oxygenation. To minimize the influence of ventilator support level on diaphragm thickness and excursion, all measurements were conducted under a stable depth of sedation.

##### 2.2.2.2 Quadriceps and rectus femoris measurements

With the patient supine and legs fully extended, the midpoint of the anterior thigh was marked. The probe was placed transversely at a point 10 cm above the superior border of the patella to obtain the image for measuring quadriceps layer thickness and rectus femoris cross-sectional area based on pragmatic and nursing-oriented considerations. In the NICU population, severe neurological impairment, limb asymmetry, edema, and positioning constraints often limit reproducible identification of classical landmarks such as 50% thigh length or ASIS–patella ratios (17). The total quadriceps muscle layer thickness (QMLT), rectus femoris thickness (RF-MLT), and cross-sectional area (RF-CSA) were recorded (13). Each parameter was measured three times and averaged.

##### 2.2.2.3 Diaphragm thickness and thickening fraction

For diaphragm DTe and DTi, the high-frequency linear probe was placed in the zone of apposition at the right mid-axillary line, between the 8th and 10th intercostal spaces. The 9th intercostal space was selected as the standard measurement window when clearly visualized. The DTF was calculated as = (DTi–DTe)/DTe × 100% (18).

##### 2.2.2.4 Diaphragm excursion

A low-frequency phased-array probe was positioned below the right costal margin, and DE was measured in M-mode during quiet breathing (19).

##### 2.2.2.5 Gastrocnemius measurements

Muscle thickness was measured at the maximal bulge of the gastrocnemius, and the pennation angle between the muscle fascicles and the aponeurosis was determined in the longitudinal plane (20).

##### 2.2.2.6 Calf circumference

Bilateral calf circumference was measured 10 cm above the superior border of the patella.

##### 2.2.2.7 Lower-limb muscle strength

Muscle strength was evaluated using the MRC scale when the patient was awake and cooperative. The flexor and extensor muscle groups of both lower limbs were assessed, yielding a total score ranging from 0 to 60, with lower scores indicating weaker muscle strength (21). The extended lower-limb MRC framework was adopted to capture bilateral and antagonist muscle involvement, which is particularly relevant in neurosurgical patients with hemiplegia or asymmetric deficits (17).

Prior to study initiation, two ICU nurses certified in critical care ultrasonography underwent standardized training and pilot imaging sessions to ensure consistent acquisition techniques. Inter-observer reliability was formally assessed before patient enrollment, demonstrating excellent agreement (Intraclass Correlation Coefficient >0.90) for all major muscle and diaphragm parameters. During the study, each measurement was performed three times and averaged, further enhancing reliability.

All ultrasound measurements were performed by the two ICU nurses certified in critical care ultrasonography. Each parameter was measured three times, and the average was used for analysis. MRC scoring was conducted independently by two trained investigators; in case of discrepancies, a third investigator adjudicated the result. To ensure measurement consistency, inter-rater reliability between the two ultrasonography-certified nurses was assessed prior to the study, showing excellent agreement (Intraclass Correlation Coefficient >0.90 for all key muscle parameters) (22).

#### 2.2.3 Nutrition-related parameters

Nutrition-related parameters were collected on ICU D1, D3, and D7, including serum albumin (Alb), total protein (TP), and the modified Nutrition Risk in mNUTRIC score.

##### 2.2.3.1 Serum albumin and total protein

Blood samples were drawn at 5:00 a.m. during routine morning sampling and analyzed by the hospital’s central laboratory using an automated biochemical analyzer. Serum albumin and total protein levels reflected the patients’ overall nutritional and inflammatory status (23).

##### 2.2.3.2 Inflammatory markers and caloric intake assessment

To comprehensively evaluate metabolic status, this study concurrently collected inflammatory markers and daily caloric intake data.

1. Inflammatory Markers (IL-6 and CRP): Blood samples for interleukin-6 (IL-6) and C-reactive protein (CRP) testing were collected by night shift nurses during routine blood draws at 5:00 AM on days 1, 3, and 7 of ICU admission. IL-6 was measured by enzyme-linked immunosorbent assay (ELISA), and CRP was determined by immunoturbidimetry on a fully automated biochemical analyzer in the department of laboratory according to the manufacturer’s instructions (Shenzhen Lifotronic Technology Co., Ltd).
2. Daily Calorie Intake Achievement Rate: Actual daily calorie intake was calculated by the bedside nursing team based on enteral/parenteral nutrition records. The daily calorie achievement rate was calculated using the following formula: (Actual Calories Consumed/Preset Target Calories) × 100%. The preset target was 25–30 kcal/kg/day. The average values from D1–D3 and D1–D7 were used for correlation analysis with concurrent muscle assessment results.

##### 2.2.3.3 mNUTRIC score

The mNUTRIC score, derived from the original NUTRIC tool with the IL-6 component removed, was used to assess nutritional risk in critically ill patients (24). The score incorporates age, APACHE II score, SOFA score, number of comorbidities, and hospital length of stay prior to ICU admission, yielding a total score from 0 to 9; scores ≥5 indicate a high nutritional risk (25). Two trained investigators independently assessed the mNUTRIC score within 24 h of ICU admission, with discrepancies resolved by a third investigator. The mNUTRIC score was selected as the primary nutritional risk assessment tool for this study because it is validated for critically ill patients and is more feasible for routine nursing assessment in clinical settings.

##### 2.2.3.4 Data collection process

All laboratory data were retrieved from the hospital’s electronic medical record and laboratory information systems. Two research nurses independently performed data extraction and verification to ensure accuracy and consistency.

To minimize potential sources of bias, we used consecutive enrollment of eligible patients during the study period. All assessments followed a standardized protocol with prespecified time points (day 1, day 3, and day 7 after enrollment). Ultrasound measurements were performed by trained operators using predefined anatomical landmarks and consistent machine settings. Outcome ascertainment at 30 days was based on review of the hospital electronic medical record system.

### 2.3 Statistical analysis

All data were tested for normality using the Shapiro–Wilk test. Continuous variables with a normal distribution were expressed as mean ± standard deviation (SD), while non-normally distributed data were presented as median and interquartile range (IQR). Categorical variables were summarized as counts and percentages.

Longitudinal changes in muscle-related and nutrition-related parameters on ICU, D1, D3, and D7 were analyzed using a linear mixed-effects model to evaluate differences across time points, and effect sizes were calculated using Cohen’s *d*.

Correlations between nutritional indicators and muscle parameters were examined using correlation analyses, with results presented as correlation coefficient matrices and heatmaps. All statistical tests were two-tailed, and *p* < 0.05 was considered statistically significant.

To assess the independent associations of nutritional/inflammatory and muscle status with clinical outcomes, multivariate regression models were employed. Negative binomial regression was used for ICU length of stay, and logistic regression for 30-day mortality and successful extubation. These models adjusted for potential confounders including age, APACHE II score, and daily sedation exposure (expressed as propofol equivalent dose).

## 3 Results

### 3.1 Baseline characteristics of patients

A total of 137 critically ill neurosurgical patients receiving mechanical ventilation were included in the study. The mean age was 57.91 ± 13.33 years, the mean body mass index (BMI) was 24.65 ± 3.80 kg/m^2^, the mean APACHE II score was 19.16 ± 6.50, and the mean SOFA score was 9.67 ± 3.27.

The primary diagnoses were intracerebral hemorrhage (*n* = 72, 52.55%), brain death (*n* = 30, 21.90%), traumatic brain injury (*n* = 26, 18.98%), and other causes (*n* = 9, 6.57%).

Among cases with hemiplegia, 10 (7.30%) had no hemiplegia, 11 (8.03%) had bilateral hemiplegia, 58 (42.34%) had left-sided hemiplegia, and 58 (42.34%) had right-sided hemiplegia.

Regarding outcomes, 78 patients (56.93%) were transferred to the rehabilitation department, 38 (27.74%) discontinued treatment, and 21 (15.33%) were discharged voluntarily. Nutritional goal achievement rates were 40.88% (*n* = 56) on day 3 and 51.09% (*n* = 70) on day 7 (see Table 1).

**Table 1:** Baseline characteristics of the patients (*n* = 137).

Variable	Mean ± SD/*n* (%)
Age (years)	57.91 (±13.33)
BMI (kg/m^2^)	24.65 (±3.80)
APACHE II score	19.16 (±6.50)
SOFA score	9.67 (±3.27)
Primary diagnosis, *n* (%)	
Intracerebral hemorrhage	30 (21.90)
Brain death	72 (52.55)
Traumatic brain injury	26 (18.98)
Others	9 (6.57)
Hemiplegia, *n* (%)	
No hemiplegia	10 (7.30)
Bilateral hemiplegia	11 (8.03)
Left hemiplegia	58 (42.34)
Right hemiplegia	58 (42.34)
Clinical outcome, *n* (%)	
Transferred to rehabilitation	78 (56.93)
Treatment withdrawn	38 (27.74)
Discharged voluntarily	21 (15.33)
Nutritional goal achievement, *n* (%)	
Day 3	56 (40.88)
Day 7	70 (51.09)

BMI, body mass index; APACHE II, Acute Physiology and Chronic Health Evaluation II; SOFA, Sequential Organ Failure Assessment (SOFA) score.

### 3.2 Longitudinal changes in muscle-related parameters within 7 days after ICU admission

Changes in muscle-related parameters on Days 1, 3, and 7 after ICU admission are summarized in Table 2. Most muscle thickness parameters showed a progressive decline over time. The right quadriceps thickness was significantly lower on Day 3 and Day 7 compared with Day 1 (D1–D3 difference = 0.247 cm, Cohen’s *d* = 0.569; D1–D7 difference = 0.188 cm, Cohen’s *d* = 0.433; both *p* < 0.05), indicating a moderate effect size.

**Table 2:** Longitudinal changes in muscle-related parameters within 7 days after ICU admission (*n* = 137).

Muscle-related parameter	D1	D3	D7	D1–D3		D1–D7		D3–D7	
				Diff	Cohen’s *d*	Diff	Cohen’s *d*	Diff	Cohen’s *d*
Right quadriceps thickness	3.40 (1.03)	3.16 (1.17)	3.22 (1.17)	0.247***	0.569	0.188**	0.433	−0.059	−0.137
Right DTi	0.25 (0.09)	0.23 (0.07)	0.22 (0.06)	0.020**	0.399	0.033***	0.664	0.013	0.265
Right DTe	0.22 (0.08)	0.20 (0.06)	0.18 (0.06)	0.021***	0.459	0.044***	0.970	0.023***	0.511
Right DTF	23.55 (10.81)	17.11 (8.96)	14.81 (7.83)	6.441***	0.749	8.740***	1.017	2.298	0.267
Right DE	1.20 (0.69)	1.18 (0.57)	1.09 (0.56)	0.019	0.040	0.110	0.231	0.091	0.191
Right rectus femoris cross-sectional area (RF-CSA)	2.58 (0.93)	2.41 (1.08)	2.43 (0.99)	0.171***	0.497	0.142**	0.414	−0.028	−0.082
Right rectus femoris thickness (RF-Th)	1.74 (0.42)	1.63 (0.51)	1.67 (0.59)	0.111***	0.491	0.068*	0.303	−0.042	−0.188
Left rectus femoris cross-sectional area (RF-CSA)	2.53 (1.03)	2.39 (0.95)	2.39 (0.97)	0.138**	0.365	0.138**	0.364	0.000	−0.001
Left rectus femoris thickness (RF-Th)	1.77 (0.53)	1.76 (0.54)	1.68 (0.50)	0.009	0.041	0.098***	0.439	0.089**	0.398
Left quadriceps thickness	3.56 (1.18)	3.39 (1.11)	3.36 (1.21)	0.164*	0.337	0.200**	0.410	0.036	0.073
Right gastrocnemius thickness	1.41 (0.35)	1.41 (0.36)	1.39 (0.36)	0.003	0.021	0.027	0.174	0.024	0.153
Right gastrocnemius pennation angle	25.57 (5.31)	26.32 (5.83)	25.26 (4.96)	−0.752	−0.196	0.314	0.082	1.066	0.277
Left lower-limb circumference	50.28(6.12)	49.89(5.85)	49.23(5.54)	0.391***	0.588	1.051***	1.581	0.661***	0.994
Right lower-limb circumference	50.48 (6.06)	49.97 (5.91)	49.38 (5.58)	0.511***	0.769	1.102***	1.658	0.591***	0.890

Values are expressed as mean (SD). *p < 0.05, **p < 0.01, ***p < 0.001 (compared with Day 1).

DTe, diaphragm end-expiratory thickness; DTi, end-inspiratory thickness DE, diaphragm excursion.

Similarly, the right diaphragm end-expiratory thickness gradually decreased (D1–D3 difference = 0.020 cm, Cohen’s *d* = 0.443; D1–D7 difference = 0.033 cm, Cohen’s *d* = 0.653).

The decline in diaphragm thickening fraction (DTF) was the most pronounced (D1–D3–6.44%, Cohen’s *d* = 0.749; D1–D7–8.74%, Cohen’s *d* = 1.017), However, all diaphragm ultrasound measurements in this study were intentionally performed under stable assist/control ventilation with controlled sedation, specifically to standardize ventilatory conditions and minimize variability. Under these conditions, we acknowledge that diaphragm thickening fraction and excursion primarily reflect ventilator-driven displacement rather than active voluntary contractility. These parameters are physiologically limited under controlled ventilation and should not be equated with spontaneous diaphragmatic effort.

The cross-sectional area of the rectus femoris also decreased significantly from D1 to D3 and D7, showing a moderate effect size.

MRC score decreased notably within the first week, with the left leg (difference = 0.931, Cohen’s *d* = 0.661) and right leg (difference = 1.102, Cohen’s *d* = 1.102) demonstrating moderate-to-large effect sizes, indicating evident muscle weakness.

### 3.3 Longitudinal changes in nutritional parameters during the first 7 days of ICU stay

Nutritional parameters were assessed on days 1, 3, and 7 after ICU admission. Serum albumin and total protein levels remained relatively stable across the three time points, with no statistically significant differences and small effect sizes.

In contrast, the mNUTRIC score showed a progressive upward trend over time, with significant increases observed from D1 to D7 (Cohen’s *d* = −0.285, *p* < 0.05) and from D3 to D7 (Cohen’s *d* = −0.365, *p* < 0.01). The IL-6 score increased from D1 to D3 but decreased significantly by D7 (Cohen’s *d* = 0.658, *p* < 0.001). CRP score increased significantly from D1 to D3 (Cohen’s *d* = −1.158, *p* < 0.001) and then decreased from D3 to D7 (Cohen’s *d* = 0.603, *p* < 0.001). The net change from D1 to D7 was also an increase (Cohen’s *d* = −0.555 *p* < 0.001). The daily calorie intake achievement rate score showed a progressive upward trend over time, with significant increases observed during the D1 to D3 period (Cohen’s *d* = −2.227, *p* < 0.001), D1 to D7 period (Cohen’s *d* = −3.251, *p* < 0.001), and D3 to D7 period (Cohen’s *d* = −1.025, *p* < 0.001) (see Table 3).

**Table 3:** Longitudinal changes in nutritional parameters during the first 7 days of ICU stay (*n* = 137).

Nutritional parameters	D1	D3	D7	D1–D3		D1–D7		D3–D7	
				Diff	Cohen’s *d*	Diff	Cohen’s *d*	Diff	Cohen’s *d*
Serum albumin	30.05 (4.62)	30.26 (2.68)	29.97 (3.39)	−0.206	−0.056	0.081	0.022	0.287	0.079
Total protein	51.84 (7.93)	52.87 (5.55)	53.48 (6.18)	−1.027	−0.176	−1.638	−0.281	−0.611	−0.105
mNUTRIC	4.61 (1.25)	4.53 (1.15)	4.90 (1.14)	0.080	0.085	−0.285*	−0.300	−0.365**	−0.385
IL-6	131.19 (112.10)	142.49 (107.56)	68.48 (55.72)	−11.304	−0.119	62.712***	0.658	74.017***	0.777
CRP	56.24 (45.17)	109.10 (50.01)	81.59(54.28)	−52.862***	−1.158	−25.345***	−0.555	27.516***	0.603
Daily calorie intake achievement rate	31.46 (8.34)	63.84 (21.07)	80.30 (10.13)	−32.435***	−2.227	−47.363***	−3.251	−14.928***	−1.025

Values are expressed as mean (SD). RF-CSA, rectus femoris cross-sectional area; RF-Th, rectus femoris thickness; DTF, diaphragm thickening fraction; DE, diaphragm excursion; IL-6, interleukin-6; CRP, C-reactive protein.

*p < 0.05, **p < 0.01, ***p < 0.001 (compared with day 1).

### 3.4 Correlation analysis between nutritional parameters and muscle-related parameters

**Figure 2:**
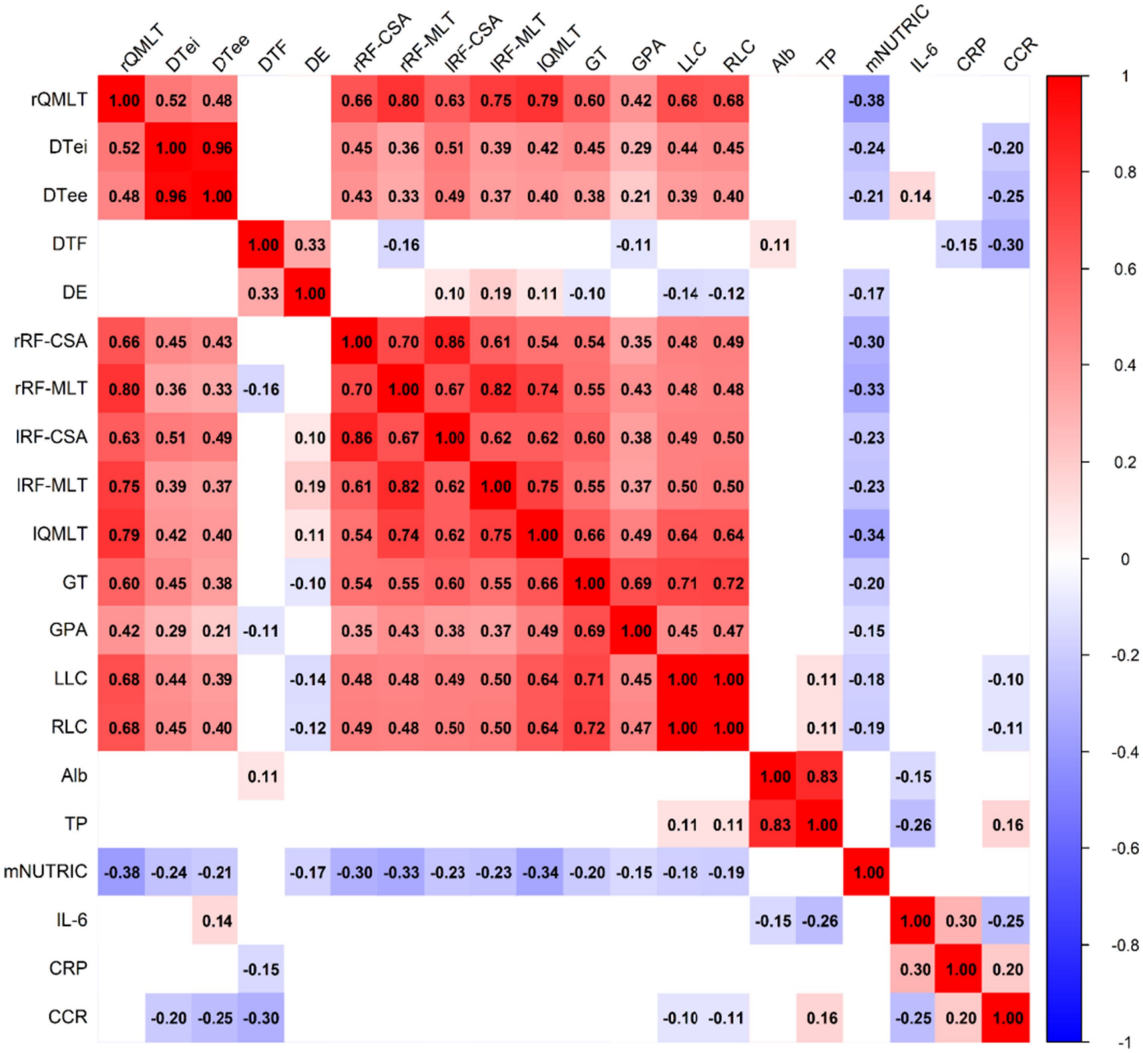
Correlation between nutritional parameters and muscle-related parameters in ICU patients (*n* = 137).

The correlation analysis results are presented in Figure 2. Except for right diaphragm hypertrophy, the mNUTRIC score was significantly negatively correlated with all 13 muscle-related parameters, including right quadriceps thickness, right DTi, right DTe, DTF, DE, right RF-CSA, right RF-Th, left RF-CSA, left RF-Th, left quadriceps thickness, right gastrocnemius thickness, right gastrocnemius pennation angle, and bilateral lower-limb circumference (all *p* < 0.05).

In contrast, serum albumin and total protein were only significantly positively correlated with diaphragm thickening fraction and bilateral lower-limb circumference (*p* < 0.05), indicating that these biochemical nutritional parameters reflected changes in some, but not all, muscle functions.

Overall, these findings suggest that increased nutritional risk is closely associated with muscle mass loss, whereas better protein levels are associated with preserved muscle function. However, since all diaphragm ultrasound measurements in this study were intentionally performed under stable assist/control ventilation with controlled sedation, increased nutritional risk cannot be considered associated with diaphragmatic dysfunction.

### 3.5 Dimensionality reduction analysis of nutritional and inflammatory markers and muscle parameters

Analysis of multicollinearity among the six nutritional and inflammatory indicators reveals that the VIF values for Alb and TP exceed 3, while the remaining indicators are below 2. This indicates moderate correlations exist among the six indicators, but no severe multicollinearity issues are present. To further evaluate the combined effects of different nutritional and inflammatory markers on muscle indicators and outcome measures, factor analysis was applied to reduce the dimensionality of the six markers. Factors were extracted based on eigenvalues greater than 1, using the variance-maximization rotation method. This yielded four factors explaining 89.10% of the cumulative variance, indicating that these factors adequately represent the numerical variations of the original six markers. Based on factor loadings, the four factors were defined as: protein factor (RC1), inflammation factor (RC2), calorie adequacy factor (RC3), and mNUTRIC factor (RC4). See Table 4 for details.

**Table 4:** Dimensionality reduction results of factor analysis for 6 nutritional and inflammatory markers.

Compound	RC1	RC2	RC3	RC4
SS loadings	1.837	1.286	1.220	1.006
Proportion Var	30.60%	21.40%	20.30%	16.80%
Cumulative Var	30.60%	52.00%	72.40%	89.10%

Analysis of multicollinearity among the 15 muscle metrics revealed that, except for right diaphragm increase rate, right diaphragm displacement, and right gastrocnemius feather angle, all other metrics had VIF values exceeding 3. This indicates strong correlations among the 15 metrics, suggesting severe multicollinearity issues. Factor analysis was applied to the 15 muscle metrics for dimensionality reduction. Factors were extracted based on eigenvalues >1 using the variance-maximization rotation method, yielding four factors explaining 79.90% cumulative variance. This indicates the four factors adequately represent the original 15 metrics’ numerical variations. Based on factor loadings, the four factors were defined as: thigh muscle mass factor (MRC1), muscle mass factor (MRC2), diaphragm thickness factor (MRC3), and diaphragm displacement factor (MRC4). See Table 5 for details.

**Table 5:** Dimensionality reduction results of factor analysis for 15 muscle markers.

Compound	MRC1	MRC4	MRC3	MRC2
SS loadings	4.324	3.305	2.205	1.354
Proportion var	30.90%	23.60%	15.70%	9.70%
Cumulative var	30.90%	54.50%	70.20%	79.90%

### 3.6 The relationship between muscle and nutritional changes

Linear mixed-effects models were used to analyze the associations between albumin, total protein, and mNUTRIC scores with 14 muscle-related indicators. All models controlled for the following covariates: white blood cell count (WBC), procalcitonin (PCT), IL-6, serum potassium, acute gastrointestinal injury grade (AGI), tidal volume (VT), inspired oxygen concentration (FiO_2_), positive end-expiratory pressure (PEEP), lung dynamic compliance (Cdyn), peak airway pressure (Ppeak), prokinetic agents, sedation, analgesia, antibiotics, vasoactive agents, neuromuscular blockers, enteral nutrition tolerance score, and nutritional tolerance status. Analysis revealed a significant negative correlation between right quadriceps thickness and mNUTRIC score (*β* = −0.198, *p* < 0.001). The results are presented in Table 6.

**Table 6:** Associations between albumin, total protein, and mNUTRIC scores with 14 muscle-related indicators.

Dependent	Independent variables		
	Alb	TP	mNUTRIC
Right quadriceps thickness	0.023	−0.024	−0.198***
Right DTi	0.016	0.000	−0.160**
Right DTe	0.036	−0.034	−0.133**
Right DTF	0.180*	−0.067	−0.063
Right DE	0.180*	−0.160	−0.168**
Right rectus femoris cross-sectional area (RF-CSA)	0.014	−0.063	−0.020
Right rectus femoris thickness (RF-Th)	−0.071	0.010	−0.192***
Left rectus femoris cross-sectional area (RF-CSA)	0.028	−0.072	0.046
Left rectus femoris thickness (RF-Th)	0.021	0.038	0.030
Left quadriceps thickness	0.070	−0.017	−0.110***
Right gastrocnemius thickness	0.075	−0.118*	−0.134***
Right gastrocnemius pennation angle	0.071	−0.157	0.014
Left lower-limb circumference	0.029	−0.042*	−0.004
Right lower-limb circumference	0.042	−0.064**	−0.002

RF-CSA, rectus femoris cross-sectional area; RF-Th, rectus femoris thickness; DTF, diaphragm thickening fraction; DE, diaphragm excursion; DTe, diaphragm end-expiratory thickness; DTi, end-inspiratory thickness; DE, diaphragm excursion.

*p < 0.05, **p < 0.01, ***p < 0.001.

This study employed a robust analysis using a linear mixed model, with the four extracted factors as independent variables and the following controlled variables: white blood cell count (WBC), procalcitonin (PCT), serum potassium, acute gastrointestinal injury grade (AGI), tidal volume (VT), inspired oxygen concentration (FiO_2_), positive end-expiratory pressure (PEEP), lung dynamic compliance (Cdyn), peak airway pressure (Ppeak), prokinetic agents, sedation, analgesia, antibiotics, vasoactive agents, neuromuscular blockers, enteral nutrition tolerance score, and nutritional tolerance status. The results showed that there is a significant negative correlation between calorie adequacy factor and right quadriceps thickness, presented in Table 7.

**Table 7:** Correlation between 4 dimensionality reduction nutritional and inflammatory markers with 14 muscle-related indicators.

Dependent	Independent variables			
	RC1	RC2	RC3	RC4
Right quadriceps thickness	0.021	−0.064*	−0.080**	−0.201***
Right DTi	0.032	−0.049	−0.231***	−0.161***
Right DTe	0.019	−0.026	−0.256***	−0.136**
Right DTF	0.115*	−0.085	−0.288***	−0.042
Right DE	0.035	0.044	−0.162**	−0.168**
Right rectus femoris cross-sectional area (RF-CSA)	−0.030	−0.034	−0.054*	−0.023
Right rectus femoris thickness (RF-Th)	−0.054*	−0.030	−0.013	−0.195***
Left rectus femoris cross-sectional area (RF-CSA)	−0.027	−0.009	−0.097***	0.044
Left rectus femoris thickness (RF-Th)	0.061*	−0.012	−0.034	0.026
Left quadriceps thickness	0.061*	−0.007	−0.087**	−0.106**
Right gastrocnemius thickness	−0.037	0.017	−0.069*	−0.115***
Right gastrocnemius pennation angle	−0.070	−0.022	−0.154**	0.025
Left lower-limb circumference	−0.001	0.006	−0.055***	−0.011
Right lower-limb circumference	−0.003	−0.006	−0.055***	−0.011

RF-CSA, rectus femoris cross-sectional area; RF-Th, rectus femoris thickness; DTF, diaphragm thickening fraction; DE, diaphragm excursion; DTe, diaphragm end-expiratory thickness; DTi, end-inspiratory thickness; DE, diaphragm excursion; RC1, protein factor; RC2, inflammation factor; RC3, calorie adequacy factor; RC4, mNUTRIC factor. *p < 0.05, **p < 0.01, ***p < 0.001.

### 3.7 Correlation between nutritional indicators, muscle parameters, and core clinical outcome measures

To further analyze the impact of muscle markers and nutritional inflammation markers on outcomes, the four muscle factors and four nutritional inflammation factors obtained from factor analysis on Day 7 were used as independent variables. The dependent variables included white blood cell count (WBC), procalcitonin (PCT), serum potassium, acute gastrointestinal injury score (AGI), tidal volume (VT), inspired oxygen concentration (FiO_2_), positive end-expiratory pressure (PEEP), lung dynamic compliance (Cdyn), peak airway pressure (Ppeak), prokinetic agents, sedation, analgesia, antibiotics, vasoactive drugs, neuromuscular blockers, enteral nutrition tolerance score, and nutritional tolerance status as control variables. These factors were analyzed for their influence on ICU length of stay, 30-day mortality, and successful extubation. ICU length of stay was analyzed using a negative binomial regression model, while 30-day mortality and successful extubation were analyzed using logistic regression. Results are presented in the Table 8. MRC1, MRC2, MRC4, RC1, and RC3 significantly reduced ICU length of stay (IRR < 1), whereas RC2 significantly increased ICU length of stay (IRR = 1.110, 95% CI: 1.043–1.182).

**Table 8:** Correlation between nutritional indicators, muscle parameters, and core clinical outcome measures.

Variable	ICU length of stay		Thirty-day mortality		Successful extubation	
	IRR	95% CI	OR	95% CI	OR	95% CI
MRC1	0.899	(0.845–0.957)	0.313	(0.124–0.674)	0.990	(0.457–2.101)
MRC2	0.906	(0.826–0.993)	1.185	(0.340–4.309)	20.594	(4.596–156.743)
MRC3	0.933	(0.860–1.011)	0.124	(0.021–0.533)	5.183	(1.048–30.735)
MRC4	0.901	(0.828–0.980)	0.215	(0.040–0.964)	0.982	(0.275–3.551)
RC1	0.880	(0.822–0.942)	0.324	(0.117–0.759)	1.765	(0.732–4.568)
RC2	1.110	(1.043–1.182)	2.617	(1.230–6.311)	0.722	(0.282–1.710)
RC3	0.867	(0.764–0.983)	3.196	(0.830–13.222)	5.917	(1.330–32.249)
RC4	1.083	(0.994–1.179)	4.708	(1.657–15.575)	0.237	(0.064–0.717)

## 4 Discussion

### 4.1 Early decline in muscle mass and function in neurosurgical critically ill patients under mechanical ventilation

In this study, we observed that neurosurgical critically ill patients receiving mechanical ventilation for ≥7 days exhibited significant reductions in peripheral skeletal muscle parameters—including thickness, cross-sectional area, and MRC score of the quadriceps, rectus femoris, and gastrocnemius—during the first week of ICU admission (Days 1, 3, and 7). Concurrently, key respiratory muscle indices, such as right diaphragm end-expiratory and end-inspiratory thickness, DTF, and DE, also declined markedly. These findings are consistent with the well-documented phenomenon of early muscle wasting in critically ill patients. Previous studies have reported that skeletal muscle may lose approximately 1%–2% of mass per day during the first week of ICU stay (26–28).

Furthermore, Fu et al. (29) demonstrated that mechanical ventilation itself can induce rapid atrophy of respiratory muscles, particularly the diaphragm, a condition termed ventilator-induced diaphragmatic dysfunction (VIDD). This muscle loss is likely driven by several factors. First, severe brain injury or post-surgical states are associated with hypermetabolism, systemic inflammatory response, heightened stress, and increased muscle protein catabolism (30). Second, prolonged mechanical ventilation, deep sedation, immobilization, and neurological deficits collectively reduce skeletal and respiratory muscle activity, contributing to disuse atrophy (31). Third, mechanical ventilation substantially diminishes diaphragmatic contraction, reducing fiber recruitment, altering fiber type, impairing mitochondrial function, increasing oxidative stress, and activating proteolytic pathways, ultimately leading to diaphragm atrophy (32).

Neurosurgical patients may exhibit additional distinctive features. First, patients with intracranial injury often require prolonged sedation and activity restriction, along with intracranial pressure management, further limiting muscle activity (33). Second, neurological impairments may result in early consciousness or motor deficits, delaying initiation of rehabilitation and exacerbating disuse mechanisms (34). Third, mechanical ventilation strategies, including lung-protective modes and deep ventilatory support, reduce the workload of respiratory muscles, increasing the risk of diaphragm atrophy (35). Sklar et al. (36) reported that patients with reduced diaphragm thickness within the first 36 h of mechanical ventilation experienced delayed weaning, prolonged ICU stay, and higher mortality.

From a nursing perspective, these findings indicate that neurosurgical critically ill patients on mechanical ventilation are at high risk of early muscle wasting upon ICU admission. Nursing care should not only focus on traditional life-support parameters, electrolytes, and organ function but also include evaluation of skeletal and respiratory muscle mass and function. Routine monitoring of muscle ultrasound parameters or MRC scores at standardized time points (e.g., D1, D3, and D7) can facilitate early detection of muscle loss. Considering the pivotal role of diaphragm function in weaning, nurses should collaborate with the treatment team to implement early respiratory muscle training, minimize unnecessary sedation, optimize early lower-limb mobilization, and adopt skeletal and respiratory muscle protection strategies. Bertoni et al. (37). demonstrated that early reductions in muscle mass or function are strongly associated with weaning failure, prolonged hospitalization, and ICU-acquired weakness. In the present study, we observed significant early changes in rectus femoris, quadriceps, and diaphragm parameters with moderate-to-large effect sizes, indicating that muscle loss in neurosurgical patients under mechanical ventilation begins immediately upon ICU admission rather than in the later stages. This underscores the importance of initiating muscle-protective interventions at ICU admission rather than waiting until overt weakness develops.

### 4.2 Relationship between changes in nutritional risk and muscle wasting

In the present study, we observed that neurosurgical critically ill patients undergoing mechanical ventilation for ≥7 days exhibited a progressive increase in the mNUTRIC score and a gradual decline in serum albumin and total protein levels during the early ICU period. Concurrently, peripheral skeletal muscle thickness and respiratory muscle function indices also declined significantly, suggesting a potential association between changes in nutritional risk and muscle wasting.

Indeed, malnutrition and muscle loss in critically ill patients often interact in a vicious cycle: inadequate nutritional intake and energy imbalance accelerate muscle protein breakdown, while rapid skeletal muscle atrophy further exacerbates negative nitrogen balance and functional impairment (38). Current evidence indicates that malnutrition-induced muscle loss involves three main mechanisms. First, during systemic inflammation and hypermetabolic states, muscle protein degradation pathways, including the ubiquitin–proteasome system and autophagy–lysosome system, are significantly activated, leading to muscle protein breakdown that exceeds synthesis (39). Second, insufficient energy intake forces the body to catabolize muscle to supply amino acids and energy substrates, further promoting skeletal muscle atrophy (40). Third, low serum albumin not only reflects malnutrition but may also indicate systemic inflammation and capillary leakage, impairing peripheral muscle perfusion and anabolic capacity, thereby exacerbating functional decline (41).

Several studies support a close relationship between nutritional risk and muscle wasting. In a prospective cohort, Weijs et al. (42) reported that nutritional inadequacy in ICU patients was significantly associated with reductions in muscle cross-sectional area, with both factors independently predicting poor outcomes. Puthucheary et al. demonstrated via ultrasound follow-up that critically ill patients could lose 17%–30% of rectus femoris cross-sectional area within the first week of ICU admission, with this process closely related to energy deficit and inflammation (13). Vishwas et al. (43) further indicated that patients with mNUTRIC ≥5 experienced greater muscle thickness loss and higher risk of weaning failure. A recent multicenter study also showed that low serum albumin was associated with ventilator-induced diaphragmatic dysfunction, highlighting a pathological link between inadequate nutrition and respiratory muscle impairment (44). These findings align with the results of the present study.

It is worth noting that in neurosurgical critically ill patients, the relationship between nutritional risk and muscle wasting is more complex than in general ICU populations. On one hand, patients with brain injury often have impaired consciousness and swallowing dysfunction, exacerbating nutritional deficiency (45). On the other hand, sedation and immobilization further restrict activity, predisposing patients to severe disuse atrophy (46). These factors combined lead to more synchronous and pronounced changes in nutritional risk and muscle wasting during the early phase of mechanical ventilation. From a nursing perspective, the simplicity and accessibility of mNUTRIC and biochemical parameters provide a practical tool for early identification of high-risk patients, potentially serving as a surrogate or complementary assessment to ultrasound. This supports integrating nutritional and muscle monitoring in routine care rather than evaluating each independently.

### 4.3 Potential and limitations of conventional nutritional parameters in nursing assessment

Ultrasound has emerged as an objective and dynamic tool for monitoring skeletal muscle and respiratory muscle function in critically ill patients. However, its widespread application in nursing practice remains limited. Ultrasound requires specialized training and operator expertise, and inter-institutional differences in equipment availability and personnel resources hinder routine implementation in many ICUs (47). Thus, the question—“Can nursing provide accurate assessment without ultrasound?”—has both academic and practical significance.

While our correlation analysis showed statistically significant associations between certain nutritional parameters (particularly the mNUTRIC score) and muscle decline, it is critical to interpret these findings with caution. In the complex ICU environment, factors like sepsis, inflammation, and fluid shifts (as reflected by the non-specificity of albumin) often overshadow pure nutritional status. Therefore, these conventional parameters should not be viewed as direct surrogates for muscle mass but rather as composite indicators of illness severity and metabolic stress, which are themselves strongly associated with muscle wasting. Their value lies in risk stratification rather than precise quantification of muscle loss. Previous studies have shown that patients with mNUTRIC ≥5 not only face higher nutritional risk but also exhibit greater muscle atrophy and higher rates of weaning failure, consistent with ultrasound findings (48). Similarly, low serum albumin reflects impaired muscle protein synthesis and increased inflammatory burden, often paralleling skeletal and respiratory muscle decline (49). These observations indicate that, in resource-limited or ultrasound-unavailable settings, conventional nutritional parameters can provide reliable early risk screening to identify high-risk patients.

However, conventional indicators cannot fully replace ultrasound. Serum albumin and total protein levels are influenced by inflammation, fluid resuscitation, and hepatic or renal dysfunction, and may not accurately reflect local muscle structure or function (11, 50). The mNUTRIC score reflects overall disease severity and nutritional risk rather than precise regional muscle changes. Furthermore, biochemical markers are typically measured intermittently, lacking the continuous bedside monitoring capability of ultrasound, which is essential for assessing respiratory muscle decline or guiding weaning strategies. For example, reductions in diaphragm thickness and thickening fraction are closely associated with weaning failure—information not captured by conventional nutritional measures (51).

Therefore, nursing assessment should not be considered an “either-or” choice between ultrasound and nutritional parameters. In resource-limited settings, mNUTRIC combined with albumin and total protein provides a feasible method for early identification of high-risk patients and timely implementation of nutritional and rehabilitative interventions. In resource-rich or critical decision-making scenarios—such as weaning evaluation or dynamic monitoring of rehabilitation interventions—ultrasound remains the preferred tool. Ideally, both approaches can be combined: nutritional parameters enable broad, general risk screening, while targeted ultrasound at key time points provides precise support, establishing a multi-tiered nursing assessment framework suitable for diverse ICU settings.

For neurosurgical patients under mechanical ventilation, the answer to “Can nursing provide accurate assessment without ultrasound?” is therefore nuanced. At the level of risk identification and routine care, conventional nutritional parameters play a critical role in early detection of high-risk patients. However, for weaning evaluation, rehabilitation monitoring, and scenarios requiring high-precision assessment, ultrasound remains indispensable. Future nursing practice should focus on rational allocation of assessment tools across different clinical environments to enhance applicability while maintaining precision at critical time points.

## 5 Conclusion and limitations

This study demonstrates that neurosurgical critically ill patients receiving mechanical ventilation for ≥7 days experience significant declines in muscle mass and function during the early ICU period. These changes involve not only peripheral skeletal muscles, reflected by reductions in muscle thickness and strength, but also respiratory muscles—particularly the diaphragm—exhibiting structural and functional impairment. Concurrently, patients’ nutritional risk progressively increased, with decreases in serum albumin and total protein levels, showing a certain correlation with muscle wasting. These findings suggest that conventional nutritional parameters can serve as a practical tool for risk screening, aiding nursing staff in the early identification of high-risk patients, whereas ultrasound retains an irreplaceable role in critical assessments, such as weaning evaluation and rehabilitation monitoring. Therefore, in different clinical settings, assessment tools should be allocated according to resource availability and nursing objectives: conventional nutritional parameters may serve as a broadly applicable baseline method, while ultrasound should be prioritized in critical decision-making scenarios.

Several limitations should be acknowledged. First, this study was a single-center observational study with a limited sample size, which may affect the generalizability of the results. Second, patient grouping was based on time points rather than randomized allocation, and potential confounding factors could not be fully controlled. Third, only the mNUTRIC score and serum proteins were used as nutritional indicators, without incorporating additional variables such as protein intake. Fourth, although ultrasound measurements were performed by trained investigators, operator dependence may still affect the precision of results. Fifth, precise calorie/protein intake per patient was not comprehensively documented and analyzed, potentially affecting the trajectory of muscle changes. Future studies should focus on multicenter, large-sample prospective designs, incorporating more comprehensive nutritional and functional parameters, to further validate the complementary roles of conventional indicators and ultrasound in nursing assessment.

## Data Availability

The raw data supporting the conclusions of this article will be made available by the authors, without undue reservation.
